# Arbuscular Mycorrhiza and Nitrification: Disentangling Processes and Players by Using Synthetic Nitrification Inhibitors

**DOI:** 10.1128/aem.01369-22

**Published:** 2022-10-03

**Authors:** Martin Dudáš, Petra Pjevac, Michala Kotianová, Kateřina Gančarčíková, Martin Rozmoš, Hana Hršelová, Petra Bukovská, Jan Jansa

**Affiliations:** a Institute of Microbiology, Academy of Science of the Czech Republic, Prague, Czech Republic; b Joint Microbiome Facility of the Medical University of Vienna and the University of Vienna, Vienna, Austria; c University of Vienna, Centre for Microbiology and Environmental Systems Science, Vienna, Austria; Georgia Institute of Technology

**Keywords:** ammonia-oxidizing bacteria, ammonia-oxidizing archaea, amplicon sequencing, arbuscular mycorrhiza, isotopic (^15^N) labeling and tracing, quantitative real-time PCR, *Rhizophagus irregularis*, synthetic nitrification inhibitor

## Abstract

Both plants and their associated arbuscular mycorrhizal (AM) fungi require nitrogen (N) for their metabolism and growth. This can result in both positive and negative effects of AM symbiosis on plant N nutrition. Either way, the demand for and efficiency of uptake of mineral N from the soil by mycorrhizal plants are often higher than those of nonmycorrhizal plants. In consequence, the symbiosis of plants with AM fungi exerts important feedbacks on soil processes in general and N cycling in particular. Here, we investigated the role of the AM symbiosis in N uptake by Andropogon gerardii from an organic source (^15^N-labeled plant litter) that was provided beyond the direct reach of roots. In addition, we tested if pathways of ^15^N uptake from litter by mycorrhizal hyphae were affected by amendment with different synthetic nitrification inhibitors (dicyandiamide [DCD], nitrapyrin, or 3,4-dimethylpyrazole phosphate [DMPP]). We observed efficient acquisition of ^15^N by mycorrhizal plants through the mycorrhizal pathway, independent of nitrification inhibitors. These results were in stark contrast to ^15^N uptake by nonmycorrhizal plants, which generally took up much less ^15^N, and the uptake was further suppressed by nitrapyrin or DMPP amendments. Quantitative real-time PCR analyses showed that bacteria involved in the rate-limiting step of nitrification, ammonia oxidation, were suppressed similarly by the presence of AM fungi and by nitrapyrin or DMPP (but not DCD) amendments. On the other hand, abundances of ammonia-oxidizing archaea were not strongly affected by either the AM fungi or the nitrification inhibitors.

**IMPORTANCE** Nitrogen is one of the most important elements for all life on Earth. In soil, N is present in various chemical forms and is fiercely competed for by various microorganisms as well as plants. Here, we address competition for reduced N (ammonia) between ammonia-oxidizing prokaryotes and arbuscular mycorrhizal fungi. These two functionally important groups of soil microorganisms, participating in nitrification and plant mineral nutrient acquisition, respectively, have often been studied in separation in the past. Here, we showed, using various biochemical and molecular approaches, that the fungi systematically suppress ammonia-oxidizing bacteria to an extent similar to that of some widely used synthetic nitrification inhibitors, whereas they have only a limited impact on abundance of ammonia-oxidizing archaea. Competition for free ammonium is a plausible explanation here, but it is also possible that the fungi produce some compounds acting as so-called biological nitrification inhibitors.

## INTRODUCTION

While the involvement of arbuscular mycorrhizal (AM) symbiosis in the uptake of phosphorus (P) by plants is well established, its importance for plant nitrogen (N) uptake is less frequently reported and discussed (1–4). In general, AM symbiosis establishment usually has less pronounced effects on N uptake by host plants when the primary N source is nitrates. If N is predominantly available in the form of ammonium or in poorly mobile organic N forms, and/or out of the direct reach of roots, AM symbiosis often does affect plant N uptake significantly (5–11). Among the various N forms present in soil environments, NH_4_^+^ appears to be a preferred N source for AM fungi (12–15), and strong competition between ammonia oxidizers and AM fungal hyphae for ammonium has been suggested (7, 16, 17).

Both plants and AM fungi (as well as all other living organisms) require N for their metabolism and growth, particularly as a component of nucleic acids, proteins, and a number of other molecules (3, 18). This can potentially result in highly variable effects of AM symbiosis establishment on plant N nutrition, ranging from positive to negative (19–21). The demand for and efficiency of mineral N uptake from soil by a mycorrhizal plant are generally higher than in a nonmycorrhizal plant (22, 23), resulting in lower N losses via leaching or gaseous N_2_O emissions (16, 24, 25). Thus, important feedbacks are exerted by AM symbiosis on soil processes in general and N cycling (and the relevant soil microbes) in particular. The importance of low-mobility N forms in soil (e.g., organic forms or ammonium ions) for mycorrhiza-mediated plant N nutrition has long been recognized (7, 10, 26–28). Likewise, interactions of AM fungi with symbiotic and asymbiotic N_2_ fixers (29), canonical ammonia oxidizers, and comammox and anammox species, denitrifiers, as well as microbes involved in organic N mineralization, have been investigated before (3, 16, 30, 31). However, the interactions of AM fungi with these organisms and processes within the soil N cycle remain poorly understood (16, 17), due to the complexity of the N cycle (32) and the diversity of and possible functional redundancy among the microorganisms involved. Furthermore, N_2_O emissions have usually been studied separately, so a holistic picture is still largely lacking (25).

**FIG 1:**
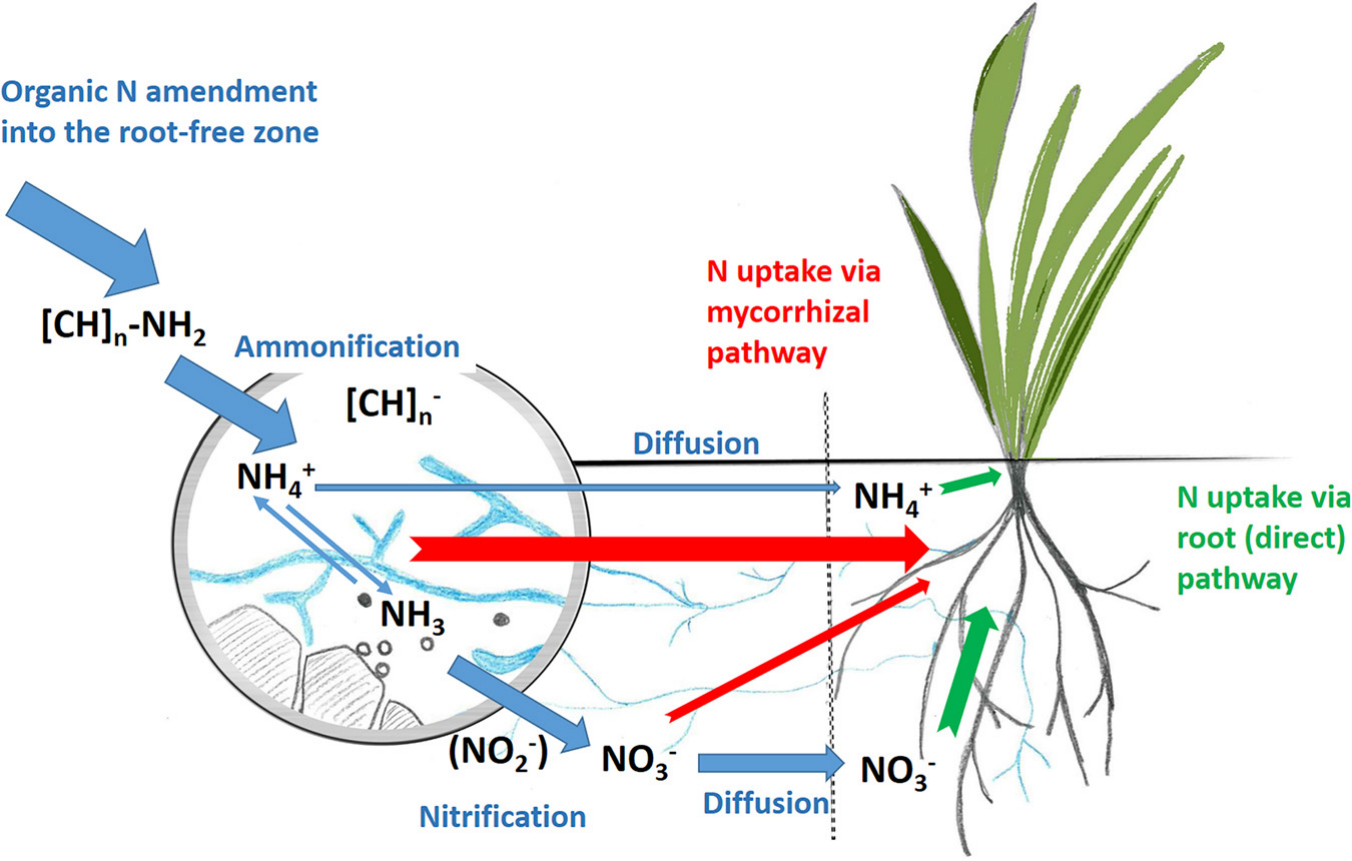
Conceptual model depicting major processes involved in nitrogen release from litter amendment and its subsequent transformation and movement in the substrate (blue arrows) and N acquisition by the plants via either the mycorrhiza (indirect) pathway (red) or the root (direct) pathway (green). Due to spatial compartmentalization of our experimental system (the dashed line illustrates the root-penetration barrier) and specific physicochemical properties (particularly, the high pH) of the substrate, which limited ammonium diffusion, the mycorrhizal hyphae had nearly exclusive access to the ammonium (NH_4_^+^) pool released from the organic amendment to a root-free substrate via microbially driven ammonification. Once ammonium was oxidized to nitrate via nitrification, N mobility in the substrate increased and roots could more efficiently acquire N directly, thus circumventing uptake via the mycorrhizal pathway. [CH]_n_ collectively represents organic C compounds after their deamination.

Here, we elucidate the role of symbiosis of plants with AM fungi in N acquisition by a model plant, Andropogon gerardii, from an organic N source (i.e., ^15^N-labeled plant litter) supplied in a soil zone beyond direct access to roots. In addition, we tested whether N uptake from litter by the plant via mycorrhizal hyphae, and associated N losses, could also be influenced by amendments with different synthetic nitrification inhibitors (SNI), such as dicyandiamide (DCD), nitrapyrin, or 3,4-dimethylpyrazole phosphate (DMPP). Particular attention was paid to the effects on abundance and community structure of aerobic ammonia-oxidizing microorganisms. The conceptual model underlying this research is presented in Fig. 1.

## RESULTS

### Plant growth and mineral nutrition.

**FIG 2:**
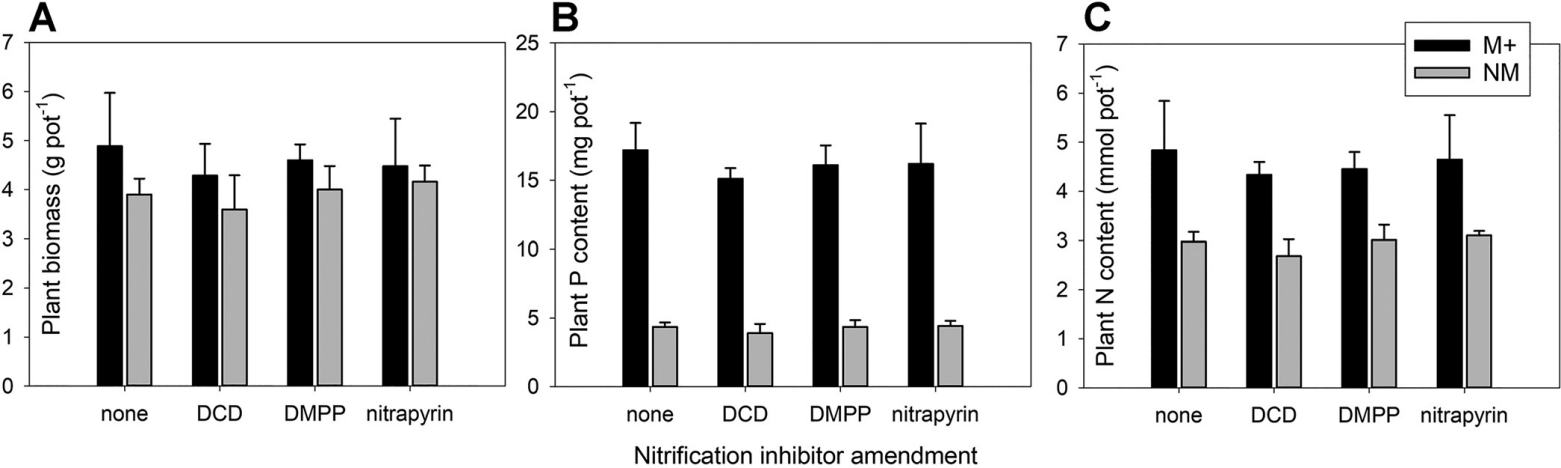
Plant biomass (shoots and roots combined, on a per-pot basis) (A) and the phosphorus (B) and nitrogen (C) contents in plant biomass as affected by mycorrhizal inoculation (M+, mycorrhizal treatment; NM, nonmycorrhizal treatment) and nitrification inhibitor amendment to the mesh bag in the root-free zone (none, DCD, DMPP, or nitrapyrin). Means and standard deviations of the means (*n* = 4) are shown. Whereas significant differences were encountered between M+ and NM treatments in all three cases, nitrification inhibitors had no significant effect on either of the variables (see Table S9 for ANOVA results).

The shoot and root biomass produced by mycorrhizal plants was significantly larger than that of nonmycorrhizal plants (Fig. 2; also, see Table S9 in the supplemental material), whereas no difference between the mycorrhizal and nonmycorrhizal plants was observed for the root-to-shoot biomass ratio (Table S9). None of the plant biomass parameters was significantly affected by the application of SNI or by the interaction between mycorrhiza formation and the SNI (Table S9). The mycorrhizal benefits (i.e., higher values in mycorrhizal than nonmycorrhizal treatments) seen in plant P and N concentrations and plant P and N contents were also significant and even stronger than the effects on plant biomass, with a particularly strong effects of AM symbiosis formation on plant P nutrition (Fig. 2; Table S9). Mycorrhizal symbiosis effectively multiplied the P content of mycorrhizal plants by a factor of four (*P < *0.001) compared with nonmycorrhizal treatment (Fig. 2; Table S9). Plant N content improved by approximately 40% (*P < *0.001) in response to the establishment of mycorrhizal symbiosis (Fig. 2; Table S9). The application of SNI and the interaction between mycorrhiza formation and SNI application did not significantly affect any parameter of plant mineral (P or N) nutrition (Table S9).

### ^15^N allocation to plants and substrates.

**FIG 3:**
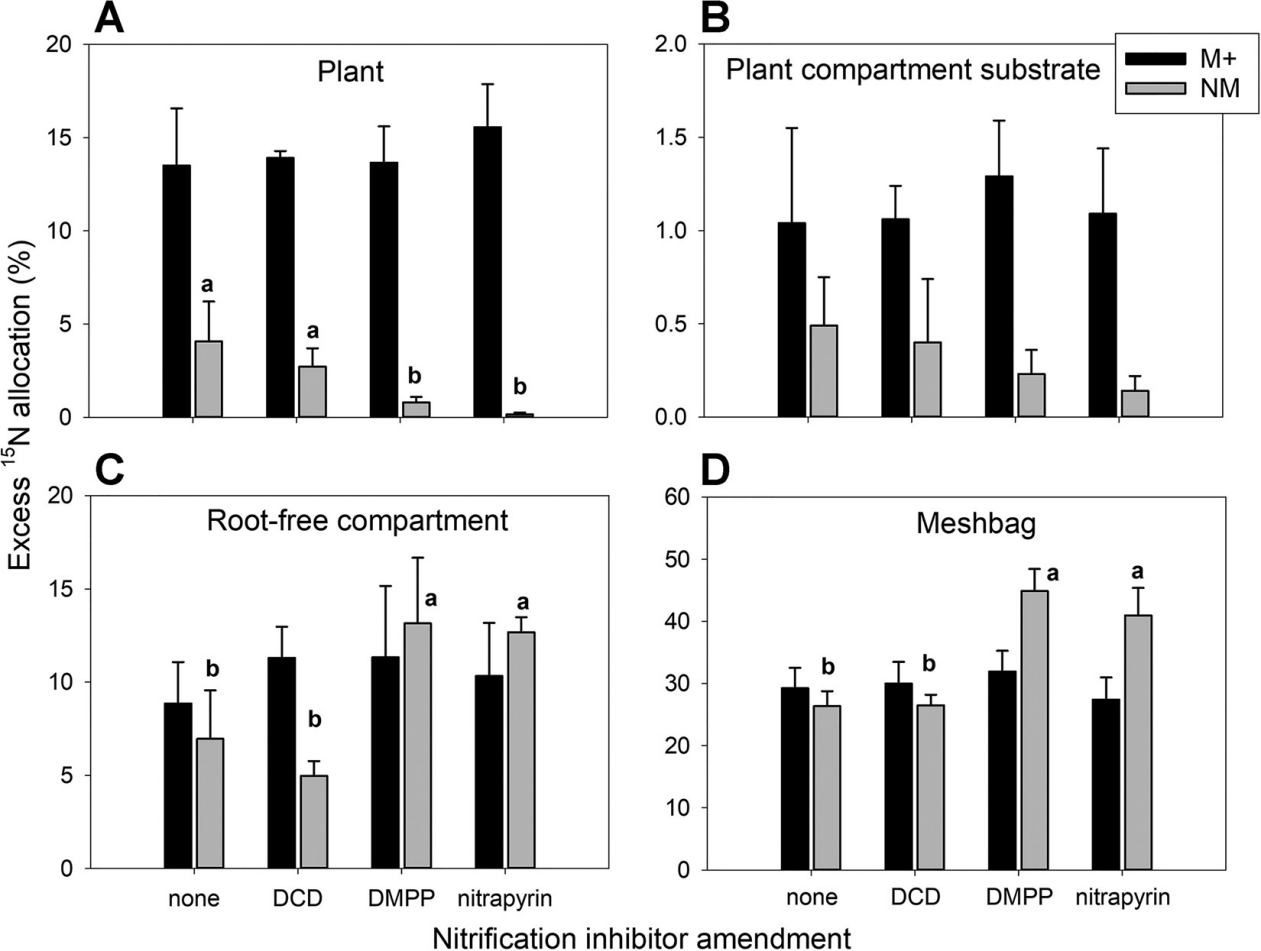
Allocation of ^15^N, added to the pots with ^15^N-labeled litter contained in the mesh bag, to different pot compartments as affected by mycorrhizal inoculation (M+, mycorrhizal treatment; NM, nonmycorrhizal treatment) and nitrification inhibitor amendment to the mesh bags (none, DCD, DMPP, or nitrapyrin). Means and standard deviations of the means (*n* = 4) are shown. Different lowercase letters within each panel distinguish significant differences between nitrification inhibitors treatments for the NM pots. Absence of letters indicates no significant differences between nitrification inhibitor treatments for the specific variable separately within the M+ or NM group. See Table S9 for all ANOVA results.

Mycorrhizal plants took up on average, across all nitrification inhibitor treatments, approximately 14% of the ^15^N supplied to the pots with the labeled plant litter, which was significantly (*P < *0.001) more than the amount of ^15^N assimilated by the nonmycorrhizal plants (~2% on average, across all SNI treatments) (Fig. 3; Table S9). No significant differences in ^15^N transfer from the litter to the mycorrhizal plants due to SNI application were observed (Table S9). The SNI did, however, significantly (*P = *0.002) affect ^15^N transfer to the plants in nonmycorrhizal pots (Fig. 3; Table S9): the ^15^N transfer from the litter to nonmycorrhizal plants in pots amended with nitrapyrin or DMPP remained below 1%, while for DCD and the control without SNI, it was well above 2% (Fig. 3).

Significantly (*P < *0.001) more ^15^N was detected in the substrate of the plant compartment of mycorrhizal pots (~1%) than in the nonmycorrhizal pots (~0.3%, on average, across all SNI treatments); no significant effect of the SNI was detected on the amount of excess ^15^N in the plant compartment (Fig. 3; Table S9). In contrast, the allocation of ^15^N from the litter to the substrate of the root-free compartment was not significantly affected by mycorrhizal inoculation (Table S9), but a significant effect of SNI application was observed across both mycorrhizal treatments, and particularly in the nonmycorrhizal pots (Table S9; Fig. 3). A significantly (*P < *0.001) higher ^15^N allocation to the substrate of the root-free compartment was observed in the DMPP- and nitrapyrin-amended nonmycorrhizal pots than in those amended with DCD or those without SNI (Fig. 3; Table S9). Finally, in nonmycorrhizal pots, a significantly (*P < *0.001) larger fraction of the ^15^N excess (~35% on average, across all SNI treatments) remained in the mesh bags than in mycorrhizal pots (~30% on average; Table S9). The ^15^N retention in the mesh bags of nonmycorrhizal pots was additionally impacted by SNI treatment and was significantly higher in DMPP- and nitrapyrin-amended pots than in DCD-amended or control pots (Fig. 3; Table S9).

In summary, calculated ^15^N losses were significantly (*P < *0.001) higher in the nonmycorrhizal pots (54%) than in the mycorrhizal pots (45%; Table S9), with a significant (*P < *0.001) effect of SNI application on ^15^N losses being observed only for nonmycorrhizal pots: calculated ^15^N losses exceeded 60% in the nonmycorrhizal pots amended with DCD or those without any SNI, whereas they remained below 50% on average for nonmycorrhizal pots amended with nitrapyrin or DMPP (Table S9).

### Mycorrhizal colonization under nitrification inhibitor treatment.

Using qPCR targeting the nuclear large ribosomal subunit gene of Rhizophagus irregularis, no significant differences in *R. irregularis* abundances were detected between treatments with different SNI in the mycorrhizal pots, either in the plant roots or in any of the substrate compartments (Table S9). The results were essentially the same when qPCR targeted the mitochondrial large ribosomal subunit gene of *R. irregularis* in the substrate DNA samples, i.e., using the mt5 primers and hydrolysis probe (see Table S8 for the data). No development of mycorrhizal fungi was detectable in the nonmycorrhizal pots (Table S8).

### Microbial guild abundances.

**FIG 4:**
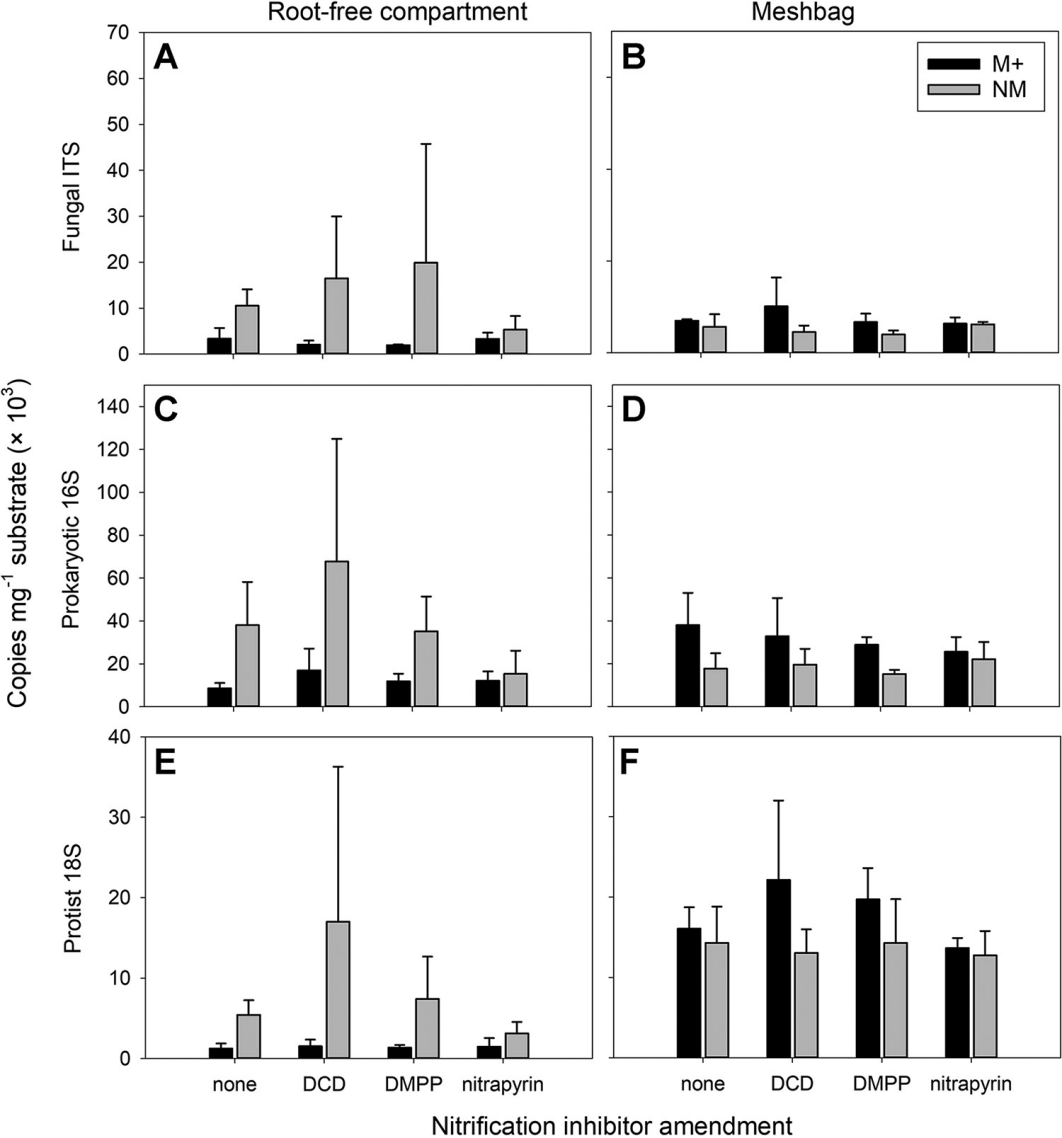
Abundance of fungi (A and B), prokaryotes (C and D), and protists (E and F), assessed by qPCR with group-specific primers in the root-free zone of the pots (A, C, and E) and in the mesh bags (B, D, and F), as affected by mycorrhizal inoculation (M+, mycorrhizal treatment; NM, nonmycorrhizal treatment) and nitrification inhibitor amendment to the mesh bags (none, DCD, DMPP, or nitrapyrin). Means and standard deviations of the means (*n* = 4) are shown. No statistically significant differences were detected between nitrification inhibitor treatments in the M+ and NM groups for any of the displayed variables. See Table S9 for all ANOVA results.

Abundances of fungi, bacteria, archaea (the last two groups were assessed together by using primers 515IL and 806IL), and protists were determined by group-specific qPCR assays (Table 1). In the substrate from plant compartments, abundance of none of these groups was affected by AM symbiosis formation (Table S9). In the root-free compartment, a consistent and statistically significant (*P < *0.05) decrease in all above-mentioned microbial guilds was observed due to AM symbiosis, while on the other hand, mycorrhizal symbiosis formation resulted in a consistent and statistically significant (*P < *0.05) increase in the same communities in the mesh bags (Fig. 4; Table S9). No significant effect of SNI application on these microbial guilds was observed in any of the pot compartments (Table S9). The results obtained with primers 515IL and 806IL were essentially the same as those obtained with primers Eub 338 and Eub 518 (see Table S8 for data), and thus, only the former are presented here.

**TABLE 1:** qPCR and endpoint PCR primers and hydrolysis (TaqMan) probes used in this study

Target	Application(s)	Name	Forward and reverse primers (5′→3′) and TaqMan probe (if applicable)^*a*^	Reference or source
16S rRNA gene of AOB	qPCR	CTO	CTOF, equimolar mixture of CTO189f (A), CTO189f (B), CTO189f (C); GGAGAAAAGCAGGGGATCG (A); GGAGGAAAGCAGGGGATCG (B); GGAGGAAAGTAGGGGATCG (C); CTOR, CTAGCYTTGTAGTTTCAAACGC (CTO654r)	93
16S rRNA gene of bacteria	qPCR	Eub	Eub 338, ACTCCTACGGGAGGCAGCAG; Eub 518, ATTACCGCGGCTGCTGG	94, 95
Internal transcribed spacer 1 region within the rRNA operon of fungi	qPCR	H	ITS0F, ACTTGGTCATTTAGAGGAAGT; 5.8S, CGCTGCGTTCTTCATCG	96, 97
28S rRNA gene of *Rhizophagus irregularis*	qPCR	intra	intraF, TTCGGGTAATCAGCCTTTCG; intraR, TCAGAGATCAGACAGGTAGCC; intraProbe, FAM-TTAACCAACCACACGGGCAAGTACA- BHQ1	91
Mitochondrial large ribosomal subunit of *Rhizophagus irregularis*	qPCR	mt5	mt5F, TTTTAGCGATAGCGTAACAGC; mt5R, TACATCTAGGACAGGGTTTCG; mt5Probe, FAM-AAACTGCCACTCCCTCCATATCCAA-BHQ1	98
V4 region of 18 rRNA gene of protists	qPCR, NGS	V4	V4F-IL, TCGTCGGCAGCGTCAGATGTGTATAAGAGACAGNNNNNCCAGCASCYGCGGTAATTCC; V4R-ILGTCTCGTGGGCTCGGAGATGTGTATAAGAGACAGNNNNNACTTTCGTTCTTGATYRA	99 (modified)
Internal DNA standard	qPCR	ISC	ISCF, CGAACCTGGACTGTTATGATG; ISCR, AATAAACAATCCCCTGTATTTCAC; ISCProbe, FAM-CACCAGGCACCAACAACGACCATT-BHQ1	91
V4 region of the 16S rRNA gene of prokaryotes	qPCR, NGS	Prokaryotic 515-806	515-IL, TCGTCGGCAGCGTCAGATGTGTATAAGAGACAGNNNNNGTGYCAGCMGCCGCGGTAA; 806-IL, GTCTCGTGGGCTCGGAGATGTGTATAAGAGACAGNNNNNGGACTACNVGGGTWTCTAAT	100
Bacterial ammonia monooxygenase	qPCR, NGS	AOB	AOBF-IL, TCGTCGGCAGCGTCAGATGTGTATAAGAGACAGNNNNNGGGGTTTCTACTGGTGGT; AOBR-IL, GTCTCGTGGGCTCGGAGATGTGTATAAGAGACAGNNNNNCCCCTCKGSAAAGCCTTCTTC	101
Archaeal ammonia monooxygenase	qPCR, NGS	AOA (*Thaumarchaeota*, formerly *Crenarchaeota*)	AOAF-IL, TCGTCGGCAGCGTCAGATGTGTATAAGAGACAGNNNNNGCAGGAGACTAYATHTTCTA; AOAR-IL, GTCTCGTGGGCTCGGAGATGTGTATAAGAGACAGNNNNNGCCATCCATCTRTADGTCCA	33
Archaeal ammonia monooxygenase	qPCR	AOA (*Crenarchaeota*)	CrenamoA23f, ATGGTCTGGCTWAGACG; CrenamoA616r, GCCATCCATCTGTATGTCCA	102
Illumina i5	NGS		AATGATACGGCGACCACCGAGATCTACACmmmmmmmmTCGTCGGCAGCGTC	Illumina
Illumina i7	NGS		CAAGCAGAAGACGGCATACGAGATnnnnnnnnGTCTCGTGGGCTCGG	Illumina

a Nucleotide nomenclature follows the IUPAC code (103). FAM, fluorescein; BHQ1, fluorescence quencher; rRNA, functional RNA forming part of the ribosomes, either small subunit (16S in prokaryotes; 18S in eukaryotes) or large subunit (28S in eukaryotes); AOB, ammonia-oxidizing bacteria; AOA, ammonia-oxidizing archaea; mmmmmmmm, Nextera XT i5 index 8-bp barcodes S502 through S522; nnnnnnnn, Nextera XT i7 index 8-bp barcodes N701 through N729. Illumina: https://support-docs.illumina.com/SHARE/AdapterSeq/Content/SHARE/AdapterSeq/Nextera/DNAIndexesNXT.htm (accessed 4 August 2022).

**FIG 5:**
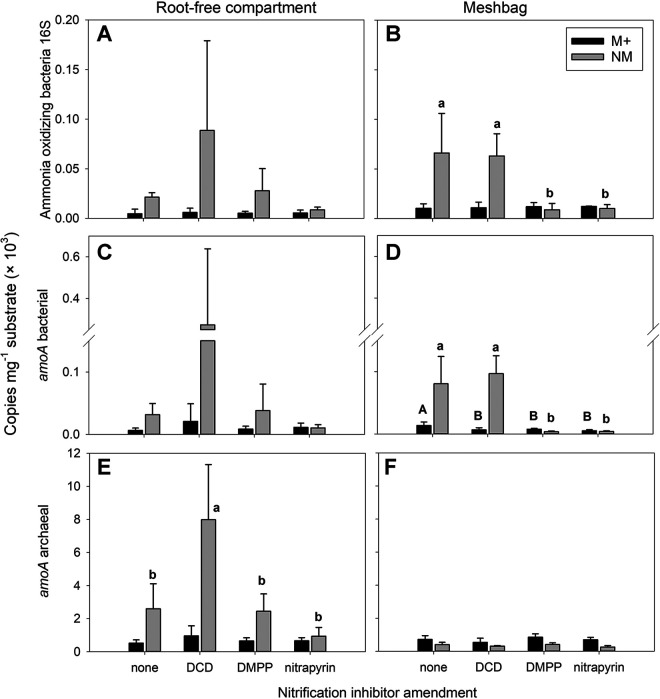
Abundance of ammonia-oxidizing bacteria assessed by qPCR targeting either 16S rRNA (A and B) or *amoA* genes (C and D) and ammonia-oxidizing archaea targeting the *amoA* gene as per Alves et al. (33) (E and F), in the root-free zone of the pots (A, C, and E) and in the mesh bags (B, D, and F), as affected by mycorrhizal inoculation (M+, mycorrhizal treatment; NM, nonmycorrhizal treatment) and nitrification inhibitor amendment to the mesh bags (none, DCD, DMPP, or nitrapyrin). Means and standard deviations of the means (*n* = 4) are shown. Different uppercase letters distinguish significant differences between nitrification inhibitor treatments in the M+ pots. Different lowercase letters distinguish significant differences between nitrification inhibitors treatments in the NM pots for each panel. Absence of letters indicates no significant differences between nitrification inhibitor treatments for the specific M+ or NM group. See Table S9 for all ANOVA results.

The effects of both mycorrhizal inoculation and SNI application on the ammonia-oxidizing communities (assessed by targeting either the 16S rRNA or *amoA* genes) remained limited in the plant compartment, with one notable exception: the abundance of bacterial *amoA* genes was significantly (*P = *0.015) and consistently reduced by mycorrhizal inoculation (Table S9). In the root-free compartment, the abundance of 16S rRNA genes of ammonia-oxidizing bacteria (AOB) and archaeal *amoA* genes, assessed using the previously described primers (33), decreased significantly (*P < *0.05) due to mycorrhizal inoculation (Fig. 5; Table S9). Moreover, the abundance of archaeal *amoA* genes in the root-free compartment was higher in the DCD-supplemented nonmycorrhizal pots than in the other nonmycorrhizal pots (Fig. 5; Table S9), resulting in the effect of the SNI applications as well as the interaction between the factors (mycorrhiza and the SNI) both being significant (Table S9). The results of archaeal *amoA* gene abundances obtained with two independent primer sets (see Table 1 for technical details and Table S8 for data) were essentially identical, and thus, only the results obtained with primers described by Alves et al. (33) are discussed here.

In the mesh bags, the AOB 16S rRNA and AOB *amoA* gene abundances were lower (*P < *0.001) in mycorrhizal pots, whereas the abundance of archaeal *amoA* genes was higher (*P < *0.001) than in the nonmycorrhizal pots (Fig. 5; Table S9). The SNI mainly had an effect on the AOB community abundance in the nonmycorrhizal pots: decreased (*P < *0.01) abundances of AOB 16S rRNA and *amoA* genes were detected in DMPP- and nitrapyrin-treated nonmycorrhizal mesh bags compared to DCD-amended mesh bags or mesh bags without any SNI (Fig. 5). A similar, although weaker, effect on the abundance of AOB *amoA* genes was also detected in mycorrhizal mesh bags (Fig. 5; Table S9). In contrast to results for AOB, The SNI did not significantly affect the abundance of *amoA* genes of ammonia-oxidizing archaea (AOA) in the mesh bags (Fig. 5; Table S9).

### Microbial community composition.

**FIG 6:**
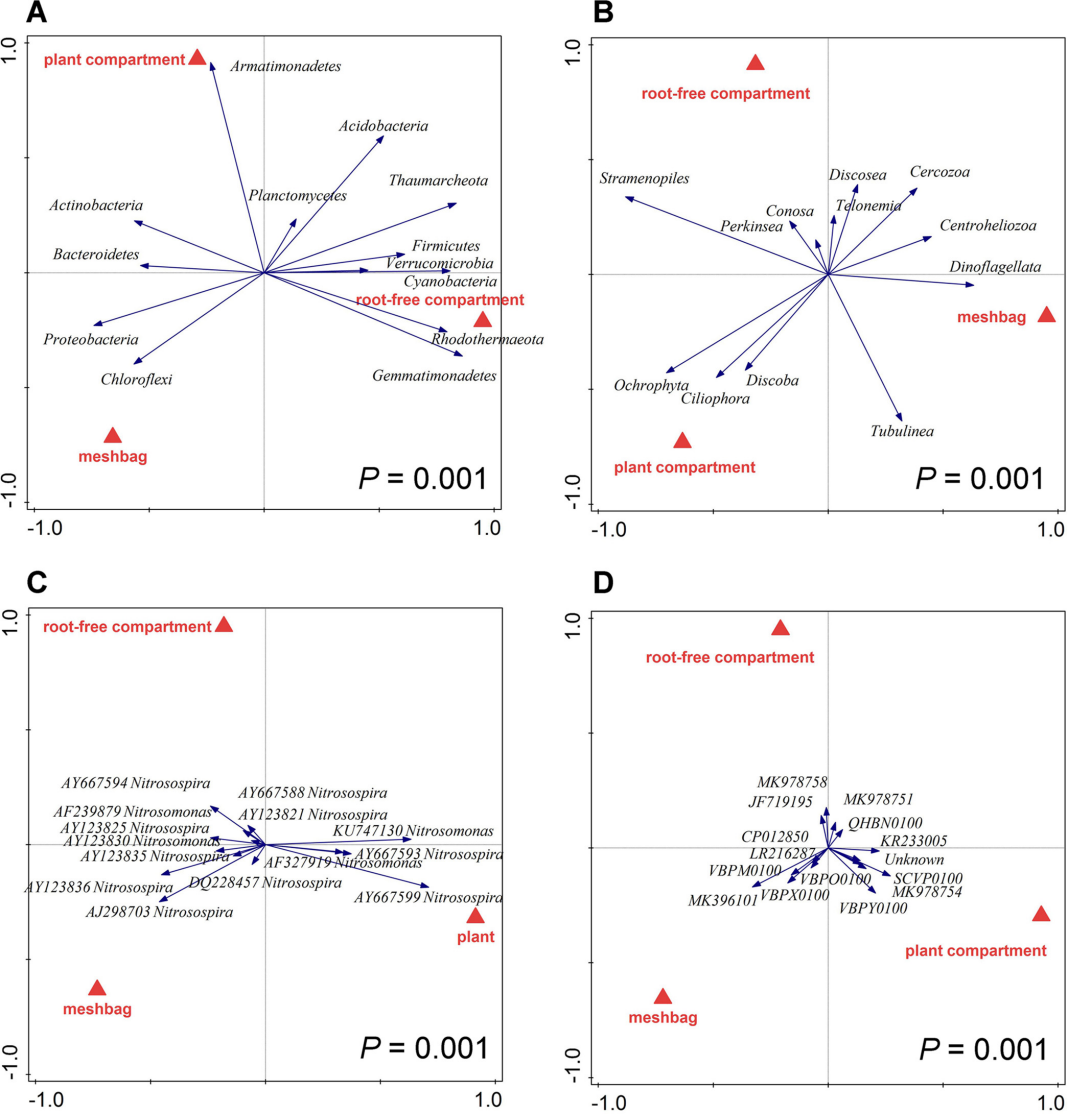
Redundancy analysis (RDA) biplots showing relationships between relative abundances of microbial taxa in four community profiles (prokaryotes [A], protists [B], ammonia-oxidizing bacteria [C], and ammonia-oxidizing archaea [D]) as affected by system compartment from which the samples were recovered (red triangles show treatment centroids). *P* values refer to partial RDA permutation tests (999 permutations) considering both canonical axes. Altogether, 96 individual substrate samples were included in the analyses (3 pot compartments, 4 nitrification inhibitors, 2 mycorrhizal inoculation treatments and 4 biological replicates for each combination of factors), except ammonia-oxidizing bacteria (C). In the latter case, sequencing depth (after sequence cleanup) in 4 individual samples was below 100 reads, and thus, the relevant pots (i.e., 12 individual samples) were excluded from the subsequent RDA analyses. See Table S10 for more details on the statistics.

The relative abundances of operational taxonomic units (OTUs) calculated from the bacterial plus archaeal 16S rRNA gene and protist 18S rRNA gene amplicons were grouped into prokaryotic and protist phyla (35 and 17 taxa, respectively). For downstream multivariate statistical analyses, a relative abundance threshold value of 1% in at least one sample was set, resulting in the inclusion of abundance data of 13 prokaryotic and 12 protistan phyla to the analyses (see Tables S4 and S5 for details). The structure of both prokaryotic and protistan communities was significantly affected by the identity of the pot compartments, indicating significant differences due to the presence of plant roots and/or patchily applied organic amendment (Fig. 6A and B; Table S10). Notably, this compartmental effect explained the vast majority (more than 50% for both prokaryotes and protists) of the data variation, whereas the other factors (i.e., addition of SNI and mycorrhizal inoculation) explained only a much smaller portion (always below 11%), if any, of the data variation for these two microbial guilds (Table S10).

**FIG 7:**
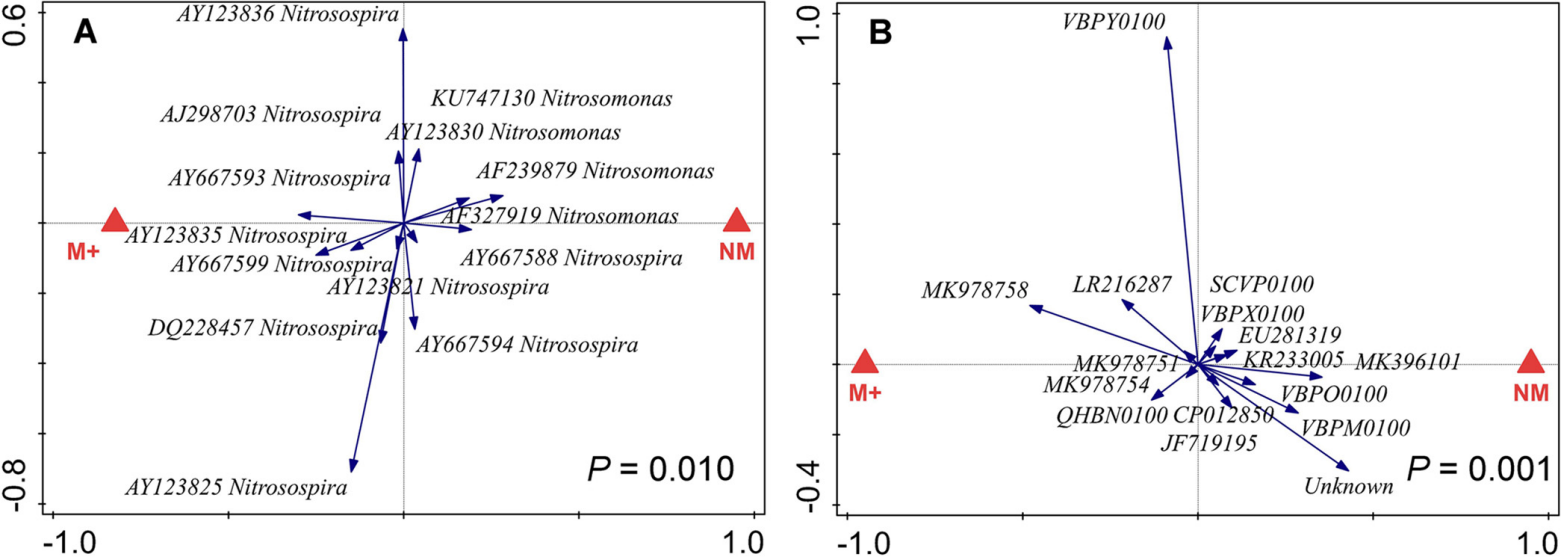
Redundancy analysis (RDA) biplots showing relationships between relative abundances of microbial taxa in two community profiles (ammonia-oxidizing bacteria [A] and ammonia-oxidizing archaea [B]) as affected by mycorrhizal inoculation of the pots from which the samples were recovered (red triangles show treatment centroids; M+, mycorrhizal treatment; NM, nonmycorrhizal treatment). *P* values refer to partial RDA permutation tests (999 permutations) considering the first (and only) canonical axis. For ammonia-oxidizing bacteria (A), 84 individual substrate samples were included (for details, see the legend to Fig. 5); for the archaea (B), 96 individual substrate samples were included (3 pot compartments, 4 nitrification inhibitors, 2 mycorrhizal inoculation treatments, and 4 biological replicates for each combination of factors). See Table S10 for more details on the statistics.

**FIG 8:**
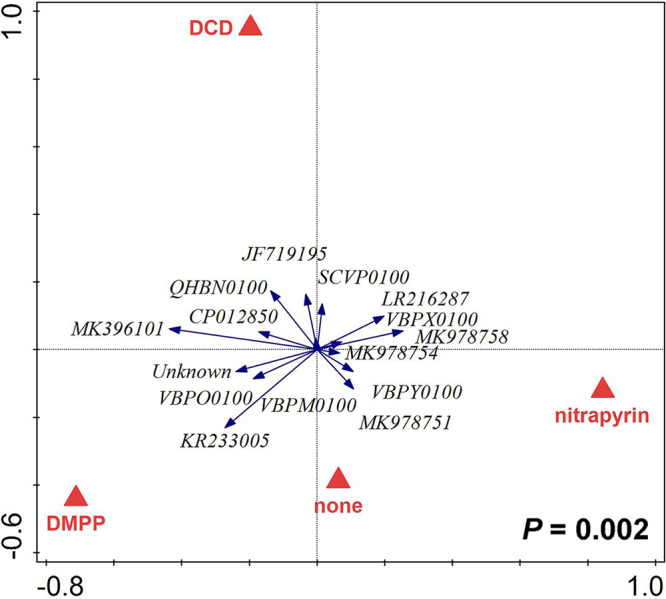
Redundancy analysis (RDA) biplot showing relationships between relative abundances of ammonia-oxidizing archaea in the community profile of substrate samples recovered from the pots, as affected by the identity of nitrification inhibitor added to the mesh bags (none, DCD, DMPP, or nitrapyrin). The *P* value refers to the partial RDA permutation test (999 permutations) considering the three canonical axes. Altogether, 96 individual substrate samples were included (3 pot compartments, 4 nitrification inhibitors, 2 mycorrhizal inoculation treatments, and 4 biological replicates for each combination of factors). See Table S10 for more details on the statistics.

For AOB *amoA* gene sequence types (21 taxa belonging to two bacterial genera), and AOA *amoA* gene sequence types (23 taxa) (see Tables S6 and S7 for details), we applied a relative abundance threshold value of 0.75% in at least one sample, resulting in 14 AOB and 15 AOA taxa included in the follow-up multivariate statistical analyses. For both *amoA* gene-carrying AOB and AOA microorganisms, both pot compartment and mycorrhizal inoculation had significant (*P < *0.05) effects on their community structures (Fig. 6C and D and Fig. 7, respectively; also, see Table S10 for more details). Moreover, the community structure of *amoA* gene-carrying archaea was significantly (*P < *0.01) affected by the application of the SNI (Fig. 8), whereas the effect of the SNI on the community structure of *amoA* gene-carrying bacteria was not significant (Table S10).

## DISCUSSION

### Mycorrhizal responses.

In the experiment described here, we observed that mycorrhizal responses were strongest for plant P uptake, followed by plant N uptake and then by plant biomass (Fig. 2), which is consistent with previous research (3, 10, 21, 34). In this experiment, significantly improved N uptake, which is not always detected in mycorrhizal experiments, was most likely due to spatially restricted root development. In contrast, the AM fungal hyphae had access to a volume of substrate several times larger than that accessible to roots, and our results agree well with previous data from similar compartmentalized experiments (7, 16, 27, 35).

### N transport from mesh bags to plants—effects of SNI and the AM fungus.

Using ^15^N isotopic labeling and tracing, we confirmed that large amounts (>10%) of ^15^N supplied as organic fertilizer in the form of clover litter were actively transported via AM fungal hyphae from the mesh bags to the plants within 42 days (Fig. 3). Furthermore, a greater portion of the supplied ^15^N isotope was retained in the plant/substrate samples from the mycorrhizal pots than in those from nonmycorrhizal pots, indicating lower N losses from the cultivation system due to mycorrhizal inoculation. This is in good agreement with previous reports (1, 24, 36, 37). Reduced losses of ^15^N from the nonmycorrhizal pots amended with DMPP or nitrapyrin compared to the nonmycorrhizal pots amended with DCD or with no SNI coincided with lower transfer of ^15^N from the mesh bags to the plants (Fig. 3). These results indicate that DMPP and nitrapyrin effectively suppressed nitrification, whereas DCD did not. By suppressing nitrification, the DMPP and nitrapyrin reduced formation of mobile N species (i.e., nitrite and nitrate) from the ^15^N-labeled litter (38). Interestingly, DCD did not suppress nitrification in our experiment, although it is a commonly applied SNI which was previously reported to be as effective as DMPP in reducing soil N losses (39). Interestingly, SNI application did not show a significant effect on the acquisition of ^15^N by mycorrhizal plants from the organic amendment. This indirectly supports the previous hypothesis that AM fungal hyphae effectively take up ammonium ions from the soil solution shortly after organic N mineralization, preventing this N from entering the nitrification pathway (16, 40, 41). Alternatively, it is possible that AM fungal hyphae themselves produced and excreted metabolites that inhibited the activity of ammonium oxidizing microorganisms by acting as biological nitrification inhibitors (BNI). Likewise, it is possible that the establishment of a mycorrhizal symbiosis stimulates production of plant root exudates with BNI activity (42).

### Microbial communities in different pot compartments.

Quantitative estimates of the abundance of bacterial and archaeal, fungal (due to selectivity of the ITS primers, this operationally defined guild is almost exclusively composed of nonmycorrhizal fungi [43]), and protistan communities revealed no effects of any of the experimental factors on the abundance of these guilds in the plant compartment. The activity and structure of microbial communities in this compartment were most likely primarily determined by rhizosphere effects (i.e., the supply of root exudates and/or root litter), which are well-recognized and strong drivers of soil life (44–46). In the root-free zone, which can be characterized as a very oligotrophic environment (a “nutrient desert”) in our experimental setup, AM fungi significantly suppressed the abundances of all microbial guilds (Fig. 4). This was most likely due to increased competition for bioavailable nutrients such as orthophosphate (47) or soluble mineral N species (41). In contrast, the abundance of the same microbial guilds was significantly higher in the organically supplemented mesh bags in the presence of AM fungi (Fig. 4), a pattern that has been reported previously (48, 49). The latter effect was most likely due to mycorrhiza-induced priming, accompanied by enhanced organic-matter decomposition supporting copious microbial communities (50–52). It is also possible that protists directly benefited by grazing the AM fungal biomass (53, 54). Alternatively, the AM fungal hyphae acting as “soil superhighways” could have facilitated the dispersion of microorganisms (bacteria, archaea, and/or protists) toward the mesh bags, as previously suggested (55, 56). However, facilitated dispersion is unlikely the main cause of the observed effect, as the same microbial communities were added to the root-free compartments and to the mesh bags at the start of the experiment. Also notable was the lack of any significant effect of the SNI on the abundance of any of the broad microbial guilds in any of the pot compartments, which strongly contrasts with the observed effects on ammonia-oxidizing microorganisms, discussed further below.

### Activities and community structures of AOA and AOB in the different pot compartments.

We observed a strong and significant negative effect of AM fungal inoculation on AOB abundance in all pot compartments. In the absence of AM fungi, DMPP and nitrapyrin treatment also resulted in decreased AOB abundance in the mesh bags, while this effect was surprisingly not observed in the DCD treatment (Fig. 5). In contrast and consistent with previous observations (57), AOA abundances were largely unaffected by the SNI (Fig. 5). Interestingly, mycorrhizal inoculation only had a significant negative effect on AOA abundance in the nutrient-limited root-free compartment, whereas a positive effect of AM fungal inoculation on AOA abundances was observed in the mesh bags (Fig. 5). The very divergent effects of AM fungi and the SNI on the abundances of AOB and AOA not only confirm the previously reported differential sensitivity of soil AOB and AOA to the SNI (58) but also point toward differential fitness of these two microbial groups in competition with AM fungi, or sensitivity to AM fungi-produced metabolites. In addition, the quantitative molecular assays indicated generally higher levels of *amoA* genes of AOA than AOB per unit weight of soil, a recurrent pattern in soil microbiome analyses (59, 60).

At first glance, these results suggest that in our experimental system, AOA play a more important role in ammonia oxidation than AOB (61–63). However, it is important to note that *amoA* gene abundances do not directly relate to the metabolic activity of the respective microorganisms or their contribution to the process of ammonia oxidation (64, 65). When these results are interpreted in accordance with the rates of ^15^N uptake by the nonmycorrhizal plants (see above), it becomes evident that the AOB were actually responsible for the majority of ammonia oxidation in our experimental system, consistent with other studies (66–68). Our results indicate that, while highly abundant, AOA are likely not particularly active at oxidizing ammonium under the given conditions.

Another interesting, and counterintuitive, observation was the increased abundance of AOA (and to a lesser extent of AOB) in the root-free zone of nonmycorrhizal pots when DCD was used as an amendment in the mesh bags. This could be related to the fact that DCD, unlike nitrapyrin and DMPP, is water soluble and thus more mobile and degrades to urea and (subsequently) ammonium within a few weeks of application (69, 70). Such degradation could have promoted nitrification in the root-free zone. It is indeed surprising that DCD was shown to be completely ineffective in our hands, despite being one of the oldest SNI studied for almost 80 years and one that is still being used regularly around the world (71). Longer-term efficiency of the different SNI has been shown to be soil and environmental context dependent (72). Notably, the efficacy of DCD was previously reported to decrease with increasing soil pH (72, 73). Consequently, the high pH of our experimental substrate (8.9 before planting [74] and 8.4 at the end of the experiment; *n* = 16) might be the main reason that DCD was rendered practically ineffective in our experimental system.

### AOA and AOB community structures.

An important result of NGS profiling of microbial communities was that AOA community composition (i.e., relative abundances of different taxa) was significantly affected by the different SNI amendments (Fig. 8), whereas AOB composition was not (Table S10). Furthermore, both pot compartment identity and mycorrhizal inoculation had significant effects on both of these ammonia-oxidizing microbial guilds (Fig. 6C and D and 7).

In our system, effective SNI (i.e., nitrapyrin and DMPP) suppressed all AOB without significant discrimination between taxa, while distinct AOA taxa responded differently to the SNI. Similar results were previously obtained regarding the sensitivity of AOB and AOA to antibiotics (75). While the AOA *amoA* gene diversity in all samples analyzed here was strongly dominated by sequences related to a recently described, cold-adapted *Nitrosocosmicus* strain, “*Candidatus* Nitrosocosmicus arcticus” (76), amendment with SNI still resulted in significant shifts in community composition (Fig. 8; Table S10). This is either due to the fact that competing AOB populations were suppressed by the SNI, allowing AOA to occupy their niches, or due to differential sensitivity of distinct AOA taxa to the supplied SNI. It was recently shown that distinct AOA, and in particular the *Nitrosocosmicus-* and *Nitrososphaera-*related AOA, which are both abundant in our experimental system (Table S7), have very different affinities for ammonia (77). Compositional shifts in the AOA community thus could be related to the proliferation of taxa with different substrate preferences in SNI-amended pots, as ammonia availability in these pots is likely altered in comparison to SNI-free controls (although we currently have no experimental data to prove this).

Either way, our results indicate there is considerable functional redundancy and versatility within the AOA communities that possibly leads to community composition shifts without necessarily changing the total abundance of the guild.

### Conclusions and future perspectives.

In summary, successful competition of AM fungi with other soil microbes for mineral nutrients in general and free ammonium in particular leads to a reduction of N losses by leaching and/or N volatilization (78–81). Our results further suggest that AM fungi are more competitive at scavenging ammonia than ammonia-oxidizing microorganisms, and AOB in particular. This competitive advantage might be driven by a higher affinity or higher reaction rate of ammonia transporters of AM fungi compared to those of AOB (and to a lesser extent AOA), as has been previously determined for other organismal groups such as phytoplankton (82, 83). However, given the periplasmic location of the ammonia monooxygenase (AMO) enzyme in both AOA and AOB, which makes the process of ammonia oxidation ammonium transport independent, and the comparatively high affinity of the AMO enzyme for ammonia (77), it is also possible that AM fungi produce and exude metabolites that directly inhibit ammonia oxidation in the vicinity of the AM fungal hyphae. The mode of competition, however, cannot be resolved from the currently available data and deserves to be further explored in detail.

Regardless of the underlying mechanism: Given the decreased N loss and increased plant N acquisition promoted by AM fungi, mycorrhizal inoculation and native AM fungal community management have to be considered as prospective tools to improve N fertilizer use efficiency, especially when the fertilizer is provided as ammonium or as organic N. Consequently, AM fungi could supplement or fully replace the application of SNI. Such compounds are widely (and successfully) used in tandem with N fertilizers to reduce N losses (84–86). Agricultural management practices that reduce nitrification rates are generally desirable, as they have the potential to attenuate emerging environmental threats such as groundwater pollution, eutrophication of surface waters, and greenhouse gas (e.g., N_2_O) emissions.

To gain more insight into these intricately interwoven processes, further specific studies aimed at elucidating the modes of interaction and competition between AM fungi and AOB/AOA, as well as the potential inhibition of AOB by AM fungi, are needed. Such studies should also include measurements of ammonium, nitrite, and nitrate levels in the different compartments and at different time points. Ideally, such studies should be further supported by culturing the various microbes under *in vitro* conditions, although some of them (particularly the soil AOA) are notoriously difficult to isolate (87).

## MATERIALS AND METHODS

### Experimental setup, establishment, and maintenance.

The experiment was conducted in tall pots (20 cm high by 11 cm wide by 11 cm deep), each containing 2 L of cultivation substrate. Each pot contained two zones, a 500-mL central cylinder and the surrounding space (1.5 L). While the first zone housed the plants and was accessible to both roots and the AM fungi (if present), the surrounding space was designed to be inaccessible to roots. Zone separation was ensured by a root-impermeable barrier, composed of a scaffold-forming plastic grid (commercially available cheese mold; no. P00718; Annelli, Montanaso Lombardo, Italy) lined with a polyamide mesh fabric (42-μm mesh size; commercially available as Uhelon 130T; Silk & Progress, Brněnec, Czech Republic). The design of the pots with respect to the establishment of the root-free zone was as in previous experiments described in more detail elsewhere (7, 8, 16).

The cultivation substrate was a mixture (45:45:10 [vol/vol/vol]) of autoclaved quartz sand, autoclaved zeolite, and gamma-irradiated (>25 kGy) soil from Litoměřice, Czech Republic. Physicochemical properties of this substrate have been described previously (74, 88). To ensure that the pots contained sufficient amounts and similar starting communities of soil microorganisms and protists, a nonmycorrhizal microbial inoculum (also called a mock inoculum), which was described in detail previously (89), was added to the substrate. The central cylinders received 2% of their volume (i.e., 10 mL) of the mock inoculum, which was suspended in 50 mL sterile water and filtered through a 20-μm steel mesh (to prevent accidental contamination with AM fungi). The substrate that filled the surrounding space was mixed with 4% (i.e., 60 mL) of solid mock inoculum without plant roots. The roots were manually removed from the inoculum before applying it to the substrate.

While the microbial mock inoculum was applied uniformly to all pots, half of the pots (pots 1 to 16) also received AM fungal inoculum, while the others (pots 25 to 40) served as nonmycorrhizal controls. AM fungal inoculum (i.e., sterile AM fungal biomass) was obtained from monoxenic cultures of *Rhizophagus irregularis* isolate LPA9, produced for 6 months in association with Cichorium intybus Ri T-DNA-transformed roots, as described previously (40). The living biomass (i.e., extraradical hyphae and spores) of the AM fungus was released from the solid cultivation medium by incubation in citrate buffer (10 mM) at pH 6.0 for 30 min and wet sieving through a 20-μm steel mesh. Approximately 30,000 AM fungal spores (besides hyphal fragments) were added to each mycorrhizal pot 2 cm below the surface.

In each of the central cylinders, 50 seeds of *Andropogon gerardii* (obtained from Jelitto Staudensamen, Schwarmstedt, Germany) were sown in a layer ~1 cm below the substrate surface at the beginning of the experiment. The substrate surface was moistened twice daily, using deionized water applied with a water nebulizer. After the plants emerged above the substrate (which happened 3 days after sowing [DAS]), the pots were watered once daily with deionized water, to maintain substrate moisture at 65 to 80% of the water holding capacity (determined by weighing the pots).

**FIG 9:**
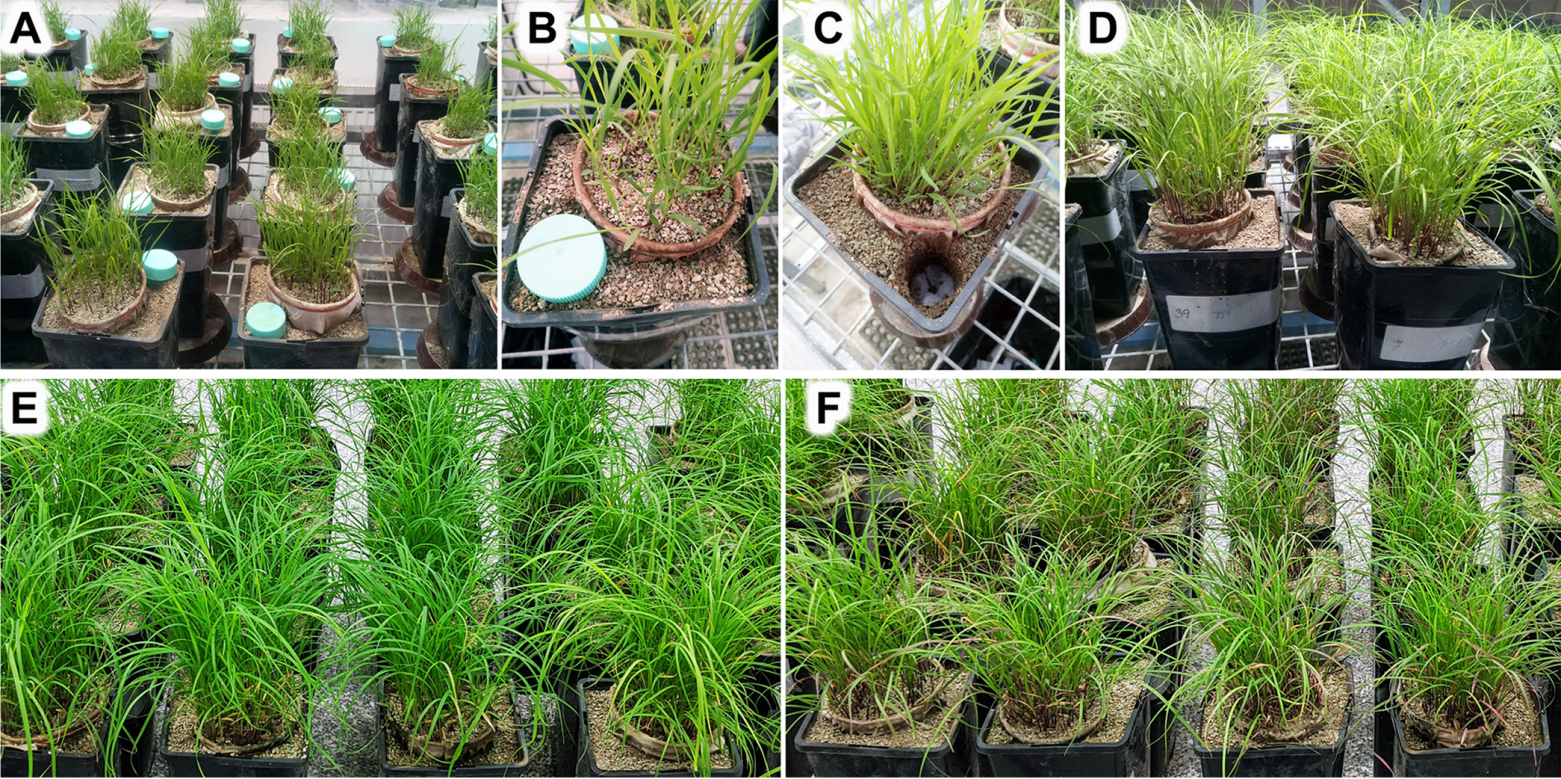
Experimental pots with the *Andropogon gerardii* plant roots confined to the central plant compartments in the glasshouse 25 (A) and 28 (B) DAS, showing the placement of empty centrifugation vials in the substrate in the root-free zone. The vials were then replaced by mesh bags (C) containing ^15^N-labeled plant litter with or without added nitrification inhibitors at 28 DAS. Appearance of the plants in randomly placed pots in the greenhouse at 52 DAS (D) and grouped mycorrhizal (E) and nonmycorrhizal (F) plants upon harvest at 70 DAS.

At sowing the seeds, a 50-mL conical centrifugation tube was placed in a corner of each pot (Fig. 9), as a placeholder to be replaced later by a mesh bag containing substrate with organic ^15^N-labeled amendment (plant litter). Throughout the experiment (70 days), the pots were incubated in the greenhouse of the Institute of Microbiology in Prague, Czech Republic. The positions of pots were completely randomized with respect to experimental treatments to avoid potentially confounding spatial effects. The temperature in the greenhouse varied between 18°C and 28°C (night and day, respectively) (see Table S1 for details). Two weeks after planting, natural light was supplemented with artificial lighting (high-pressure metal halide lamps, 500 W) to ensure photosynthetically active radiation flux density of more than 200 μmol m^−2^ s^−1^ at plant level throughout the photoperiod (12 h day^−1^).

Each mesh bag (made of 100-μm nylon mesh fabric and holding 20 mL/29 g of substrate) contained 60 mg of clover biomass (plant litter), which is described in full detail elsewhere (7). The litter had N isotopic composition equaling 42 atom% ^15^N and was added either with no nitrification inhibitor or with one of the following SNI: 2 mg DCD, 0.3 mg DMPP, or 2 mg nitrapyrin. Eight mesh bags with each type of amendment were prepared, and four mesh bags of each type were then used for the mycorrhizal and the nonmycorrhizal treatments, respectively. The zip-tied mesh bags were placed in the pots at 28 DAS, replacing the centrifugation vials. At this time point, the positions of the pots in the greenhouse were also completely rerandomized. Nutrient input from the litter added with the mesh bag was 1.45 mg N and 0.1 mg P per pot (see reference 7 for further details). Starting at 35 DAS, each pot received 65 mL of Long Ashton nutrient solution with reduced P concentration (see reference 74 and Table S2 for more details), added to the central cylinder each week. Nutrient input with the nutrient solution was 54.6 mg N and 2.61 mg P per pot throughout the experiment (i.e., 10.9 mg N and 0.52 mg P weekly), with N applied predominantly in the form of nitrate.

### Harvest.

The plants were harvested at 70 DAS. Plant biomass and substrate samples were processed immediately. First, plant shoots were cut at the substrate level. Thereafter, roots were extracted from the substrate by shaking, then washed thoroughly under tap water, and rinsed briefly under deionized water. Both shoots and roots were (separately) weighed fresh, placed in paper bags, and dried in a drying cabinet with forced air circulation at 65°C for 3 days before dry weight was determined. Substrate samples were collected from the central cylinder (i.e., the plant compartment [compartment A]), the surrounding space (i.e., the root-free compartment [compartment N]), and mesh bags (compartment M). A representative sample of ~20 g fresh weight was taken from each substrate compartment in each pot, placed in a paper bag, and dried at 65°C for 3 days. Both plant and substrate samples were subsequently pulverized in an MM200 ball mill (Retsch, Haan, Germany) at 25 Hz for 2 min using two stainless steel balls (10-mm diameter) per sample, prior to all downstream analyses.

### Elemental and isotopic analyses.

For analysis of P concentration in plant biomass, samples of shoots or roots (0.1 g each) were incinerated at 550°C for 12 h, the ashes were extracted with 1 mL boiling concentrated HNO_3_, and the extracts were then made up to 50 mL with ultrapure (18.2-MΩ) water. Phosphorus concentration in the acid extracts was measured spectrophotometrically by the malachite green method (90). The N and C concentrations in the plant biomass (2-mg ground samples) and substrates (20-mg ground samples), as well as the isotopic composition of N in the same samples, were analyzed using the Flash EA 2000 elemental analyzer coupled to a Delta V Advantage isotope ratio mass spectrometer (Thermo Fisher Scientific, Bremen, Germany).

### Quantitative molecular genetic analyses.

DNA from roots (~10 mg dry powder) and substrate samples (~600 mg dry powder) was extracted using the Plant DNeasy kit (Qiagen, Venlo, Netherlands) and DNeasy PowerSoil kit (Qiagen), respectively, following the manufacturer’s recommendations. To quantify DNA extraction losses, 2 × 10^10^ copies of an internal DNA standard (91) were added to each sample prior to DNA extraction.

Molecular quantification of various microorganisms (AM fungi, bacteria, fungi, AOB, AOA, and protists) in the different samples was performed by qPCR (see Table 1 for a detailed list of assays and corresponding references). All analyses were performed using the LightCycler 480 II instrument (Roche, Rotkreuz, Switzerland). Each qPCR assay was first calibrated with the product of endpoint PCR performed with the corresponding primers on the DNA extracted from at least four different substrate samples. The amplicons produced with the same primers were pooled and purified (QIAquick PCR purification kit; Qiagen), fragment length was evaluated using electrophoresis on a 0.8% agarose gel, and DNA concentration in the amplicon samples was measured using the Quant-iT PicoGreen double-stranded-DNA (dsDNA) assay (Thermo Fisher Scientific, Waltham MA, USA) on a plate reader (Infinite 200 Pro; Tecan, Männedorf, Switzerland). Dilution series were then prepared from the amplicons and used as templates for qPCR calibration as described previously (91). The qPCR quantification was carried out in 96-well plates using a 20-μL final reaction volume. Depending on whether primer sets were designed together with TaqMan (hydrolysis) probes, which would be double labeled with fluorescein as a fluorophore and BHQ1 as a quencher, reaction mixtures were prepared using two master mixes. We used either Luna universal probe qPCR master mix (M3004; for assays including a probe) or Luna universal qPCR master mix (M3003; without a probe), both purchased from New England Biosciences (Ipswich, MA, USA). Fluorescence was recorded in the SYBR green/fluorescein color channel.

### NGS analyses.

The amplified loci (the V4 region of bacterial and archaeal 16S rRNA genes, bacterial *amoA* genes, archaeal *amoA* genes, and the V4 region of protist 18S rRNA genes) and primers used for next-generation sequencing (NGS) analyses are all listed in Table 1. All listed primers were synthesized and purified by high-performance liquid chromatography (HPLC) by Generi Biotech (Hradec Králové, Czech Republic). Library preparation consisted of two subsequent endpoint PCR amplifications. The first amplification step (35 cycles) was performed in triplicate, using a PPP master mix (Top-Bio, Vestec, Czech Republic), except for the amplification of the prokaryotic 16S rRNA gene, where the TP HS DNA-free 2× master mix from the same supplier was used instead. The products of the triplicate first-step PCRs were pooled and purified using the QIAquick PCR purification kit (Qiagen). In the second PCR (10 cycles), previous amplicons were extended using Illumina i5 and i7 adapters that carried sample-specific barcode combinations (Nextera XT dual indexes [Table 1]). The products of the second PCR were purified as described above, and amplicon length was checked by standard agarose gel electrophoresis (0.8%). An equimolar mixture of all amplicons was then prepared in a single tube based on amplicon concentrations determined with the Quant-iT PicoGreen dsDNA assay (Thermo Fisher Scientific). A final size selection of this library pool, using paramagnetic beads (Agencourt AMPure XP PCR purification kit; Beckman Coulter, Brea, CA, USA) was performed, the purified library pool was then diluted to 10 pM, denatured, and sequenced on the Illumina MiSeq platform, using proprietary 600-cycle V3 chemistry (2 × 300-bp paired-end reads) at the Joint Microbiome Facility (JMF, Vienna, Austria).

### Bioinformatic analyses.

Sequencing data were first demultiplexed to individual amplicons using bcl2fastq v2.20.0.422 (Illumina, San Diego CA, USA) with default settings. The resulting data were then processed using the software package SEED v2.1.1 (92). Forward and reverse sequence reads were paired when an overlap of 20 bp was detected, with a maximum allowed mismatch set at 3 bp/15%. Subsequently, the PCR primers (both forward and reverse) were then trimmed off the paired sequences. All sequences with an average quality score of less than 30, a per-base quality score of less than 7, and a length of less than 200 bp were removed. Sequences with any ambiguous base(s) and potentially chimeric sequences were removed using the v-search algorithm embedded in SEED. Additionally, sequences of AOB *amoA* genes were size filtered to match a 452- ± 3-bp length window. Thereafter, sequences were clustered into operational taxonomic units (OTUs) using v-search at a similarity threshold of 97%. OTUs were identified by conducting BLAST of the most abundant sequences for each of the OTUs against the NCBI NR and custom databases (containing AOB *amoA* gene sequences or AOA *amoA* gene sequences), the SILVA (prokaryotic 16S rRNA gene) database, or the PR2 (protist 18S rRNA gene) database to identify and remove nontarget sequences (e.g., chloroplasts from the prokaryotic data set or plant sequences from the protist data set). The custom databases for identification of AOA and AOB based on the *amoA* gene sequences were created by downloading all NCBI GenBank entries quoting ammonia monooxygenase for archaea and bacteria, respectively. The sequencing depths per sample were rarefied separately for each data set by random resampling of sequences to the following sequencing depths: 1,500 (AOA *amoA* gene), 2,080 (AOB *amoA* gene), 16,186 (protist 18S rRNA gene), and 31,246 (prokaryotic 16S rRNA gene) reads per sample (details in Table S3). At least 80% of the individual samples were originally sequenced with greater sequencing depth than those chosen for resampling (for details, see Table S3). The OTU relative abundances in each sample were then summed at higher taxonomic levels (e.g., sequence types, genera, families, orders, or phyla) to be used in multivariate statistical analyses. The data used for the statistical analyses are provided in Tables S4 to S7.

### Calculations and statistics.

The N and P concentrations measured in the plant tissues were used to calculate the N and P contents in the shoots and roots on a per-pot basis, by using the dry biomass of the shoots and roots per pot, respectively. Allocation of ^15^N from the labeled organic amendment into the different system compartments (i.e., plant shoots, roots, and the substrate in the plant compartment, in the root-free compartment except the mesh bag, and in the mesh bag) was calculated by considering the molar amount of N in the different system compartments and the isotopic composition of the N (expressed as atoms percent). A value of 0.37 atom% ^15^N was considered isotopic background (i.e., natural abundance) for all substrate samples, while a value of 0.366 atom% ^15^N was considered isotopic ^15^N background in plant (shoot and root) biomass samples according to our previous results (16). Nonspecific ^15^N losses from our experimental system (whether due to leaching or gaseous losses) were calculated on a per-pot basis as the difference between the excess ^15^N provided with the input of the labeled plant litter per pot (i.e., 104 μmol ^15^N) and the sum of excess ^15^N in all measured compartments per pot. The data are provided in Table S8.

The abundances of the different microbial guilds/functional genes measured by qPCR were corrected for nonspecific losses during DNA extraction for each individual sample. Losses were calculated by determining the total copy number of the internal DNA standard per sample (assessed experimentally for each extraction batch), the extraction volume (200 μL), and the amount of internal DNA standard spiked into each sample (for more details, see reference 91).

Data were analyzed by one-way or two-way analysis of variance (ANOVA) using SigmaPlot for Windows v. 13.0 (Systat Software, Inc., Palo Alto, CA, USA) after being checked for normality (Shapiro-Wilks test) and subjected to a Levene median test for variance equality. Results of the ANOVA are provided in Table S9. The microbial community data derived from the NGS analyses (provided in Tables S4 to S7) were subjected to multivariate principal component analysis (PCA) and redundancy analysis (RDA), carried out in Canoco v. 5.12 (http://www.canoco5.com/index.php); details are provided in Table S10. Because of low sequencing depth of four AOB *amoA* amplicons after removal of nontarget sequences, only 84 individual samples were included in multivariate analyses of AOB *amoA* (i.e., removing the amplicons with low sequencing depth and corresponding samples from other compartments of the same pots; see Table S3 for details). In all other multivariate statistical analyses, 96 individual samples were always included. Only taxa with at least four occurrences across the relevant data sets were included into the multivariate statistical analyses.

### Data availability.

All experimental data generated within this research and used for the different statistical analyses are provided in the supplemental material (see Tables S4–S8). For the NGS data, the paired sequences (sorted by primer group but otherwise unchanged) were deposited in the Sequence Read Archive under accession number PRJNA693256.
