# Supplementary material for: Arbuscular Mycorrhiza and Nitrification: Disentangling Processes and Players by Using Synthetic Nitrification Inhibitors
Source: Appl Environ Microbiol. 2022 Oct 3;88(20):e01369-22. doi: 10.1128/aem.01369-22 (PMC9599619; doi:10.1128/aem.01369-22)
Supplement: Supplementary file 2 — Table S2. Download aem.01369-22-s0002.pdf, PDF file, 0.07 MB [file aem.01369-22-s0002.pdf]

Table S2. Recipe for Long Ashton nutrient solution used for fertilization of plants in the research described here.

Compound	Amount per 1 liter (mg)
KNO_3	404
$CaNO_3$ 4 H_2O	944
KH_2PO_4	36*
$MgSO_4$ $7H_2O$	368
NaFe EDTA	22
$MnSO_4$ $4H_2O$	2.23
$CuSO_4$ $5H_2O$	0.25
$ZnSO_4$ $7H_2O$	0.29
H_3BO_3	3.10
NaCl	10.0
$(NH_4)6Mo_7O_{24}$ $4H_2O$	0.09

* this corresponds to 20% of P concentration compared to full strength nutrient solution after Long Ashton (1, 2)

Supplementary references

1. Hewitt EJ. 1966. Sand and water culture methods used in the study of plant nutrition. Commonwealth Agricultural Bureaux, Farnham Royal (Bucks.).
2. Smith GS, Johnston CM, Cornforth IS. 1983. Comparison of nutrient solutions for growth of plants in sand culture. New Phytol 94:537–548.
